# Evidence of multiple insecticide resistance mechanisms in *Anopheles gambiae* populations in Bangui, Central African Republic

**DOI:** 10.1186/s13071-016-1965-8

**Published:** 2017-01-13

**Authors:** Marina Lidwine Olé Sangba, Aboubakar Sidick, Renaud Govoetchan, Christian Dide-Agossou, Razaki A. Ossè, Martin Akogbeto, Mamadou Ousmane Ndiath

**Affiliations:** 1 G4 Malaria Group, Institut Pasteur of Bangui, BP 926 Bangui, Central African Republic; 2 Faculté des Sciences et Techniques, Université d’Abomey Calavi, Cotonou, Benin; 3 Centre de Recherche Entomologique de Cotonou (CREC), Cotonou, 06 BP 2604 Benin; 4 Ecole Nationale des Sciences et Techniques Agricole de Djougou (ENSTA), Université des Sciences Arts et Techniques de Natitingou (USATN), Natitingou, Benin; 5 University of Colorado Denver Anschutz Medical Campus, 13001 E 17th Pl, Aurora, CO 80045 USA; 6 Ecole de Gestion et d’Exploitation des Systèmes d’Elevage (EGESE), Université d’Agriculture de Kétou (UAK), Kétou, Benin; 7 G4 Malaria Group, Institut Pasteur of Madagascar BP 1274, Ambatofotsikely Avaradoha 101, Antananarivo, Madagascar

**Keywords:** Malaria, *Anopheles gambiae*, Insecticide resistance, Bangui, Central African Republic

## Abstract

**Background:**

Knowledge of insecticide resistance status in the main malaria vectors is an essential component of effective malaria vector control. This study presents the first evaluation of the status of insecticide resistance in *Anopheles gambiae* populations from Bangui, the Central African Republic.

**Methods:**

*Anopheles* mosquitoes were reared from larvae collected in seven districts of Bangui between September to November 2014. The World Health Organisation’s bioassay susceptibility tests to lambda-cyhalothrin (0.05%), deltamethrin (0.05%), DDT (4%), malathion (5%), fenitrothion (1%) and bendiocarb (0.1%) were performed on adult females. Species and molecular forms as well as the presence of L1014F *kdr* and *Ace-1* ^*R*^ mutations were assessed by PCR. Additional tests were conducted to assess metabolic resistance status.

**Results:**

After 1 h exposure, a significant difference of knockdown effect was observed between districts in all insecticides tested except deltamethrin and malathion. The mortality rate (MR) of pyrethroids group ranging from 27% (CI: 19–37.5) in Petevo to 86% (CI: 77.6–92.1) in Gbanikola; while for DDT, MR ranged from 5% (CI: 1.6–11.3) in Centre-ville to 39% (CI: 29.4–49.3) in Ouango. For the organophosphate group a MR of 100% was observed in all districts except Gbanikola where a MR of 96% (CI: 90–98.9) was recorded. The mortality induced by bendiocarb was very heterogeneous, ranging from 75% (CI: 62.8–82.8) in Yapele to 99% (CI: 84.5–100) in Centre-ville. A high level of *kdr-w* (L1014F) frequency was observed in all districts ranging from 93 to 100%; however, no *kdr-e* (L1014S) and *Ace-1* ^*R*^ mutation were found in all tested mosquitoes. Data of biochemical analysis showed significant overexpression activities of cytochrome P450, GST and esterases in Gbanikola and Yapele (*χ* ^2^ = 31.85, *df* = 2, *P* < 0.001). By contrast, esterases activities using α and β-naphthyl acetate were significantly low in mosquitoes from PK10 and Ouango in comparison to Kisumu strain (*χ* ^2^ = 17.34, *df* = 2, *P* < 0.005).

**Conclusions:**

Evidence of resistance to DDT and pyrethroids as well as precocious emergence of resistance to carbamates were detected among *A. gambiae* mosquitoes from Bangui, including target-site mutations and metabolic mechanisms. The co-existence of these resistance mechanisms in *A. gambiae* may be a serious obstacle for the future success of malaria control programmes in this region.

**Electronic supplementary material:**

The online version of this article (doi:10.1186/s13071-016-1965-8) contains supplementary material, which is available to authorized users.

## Background

In the Central African Republic (CAR), malaria is the major public health problem and the leading cause of death among children [1, 2]. In Bangui, the capital of CAR, malaria represents over 58% of the reasons for consultations and 54% of hospital deaths among children [3]. The mortality among children under 5 years increased from 29% in 2005 to 48% in 2009 [4] because CAR is plagued by shortages of essential drugs and logistical constraints sustained by political violence. Today, the main approaches of malaria control rely on the early detection of cases by the rapid diagnostic tests (RDTs), the prompt treatment of malaria cases with artemisinin based combination therapy (ACT) and vector control strategies. The combined actions of vector control tools, including indoor residual spraying (IRS) and insecticide-treated nets (ITN), have significantly reduced the burden of malaria in many parts of the world giving hope to elimination or pre-elimination malaria programmes [5, 6]. However, the rapid and widespread insecticide resistance represents a serious threat to the ambitious goal of malaria elimination [7].

In Africa, *Anopheles gambiae*, the major malaria vector, experiences very intense selective pressure from insecticides used in malaria vector control programmes, in particular from impregnated bed nets and indoor-residual spraying [6, 8]. This is coupled with added pressure from the heavy use of insecticides in agriculture [9]. Consequently, the emergence of resistance in natural populations of *A. gambiae* to various classes of insecticides used in malaria vector control strategies has been reported in many African countries [10]. Consequently, multiple mechanisms of resistance to insecticides have been observed in anopheline populations, including target site mutation (*kdr*, *Ace-1* ^*R*^) [11–13] and increased metabolic detoxification [overproduction of esterases, cytochrome P450 monooxygenases and glutathione-S-transferases (GST)] [14, 15].

In CAR, recent entomological investigation revealed the predominance of *A. gambiae* in Bangui with a high prevalence of the L1014F *kdr* mutation [16]. However, no data are available on the current insecticide resistance using World Health Organization (WHO) bioassays in *A. gambiae* from CAR. Today there is almost unanimous agreement that the effectiveness of vector control programmes requires in depth knowledge of the insecticide susceptibility of the malaria vectors [17]. It is important to provide information on malaria vector insecticide resistance in order to help implement effective control programmes and foresee suitable resistance management strategies [7, 10], particularly in light of the political tensions in CAR since 2013. Early detection of resistance is necessary for the implementation of rational vector control programmes. It will not be possible to have reliable information without a regular and tight mapping of the resistance status of mosquitoes. This paper, therefore, reports for the first time in CAR the level, type and insecticide resistance mechanisms in *A. gambiae* populations collected in seven districts of Bangui.

## Methods

### Study sites

**Fig. 1:**
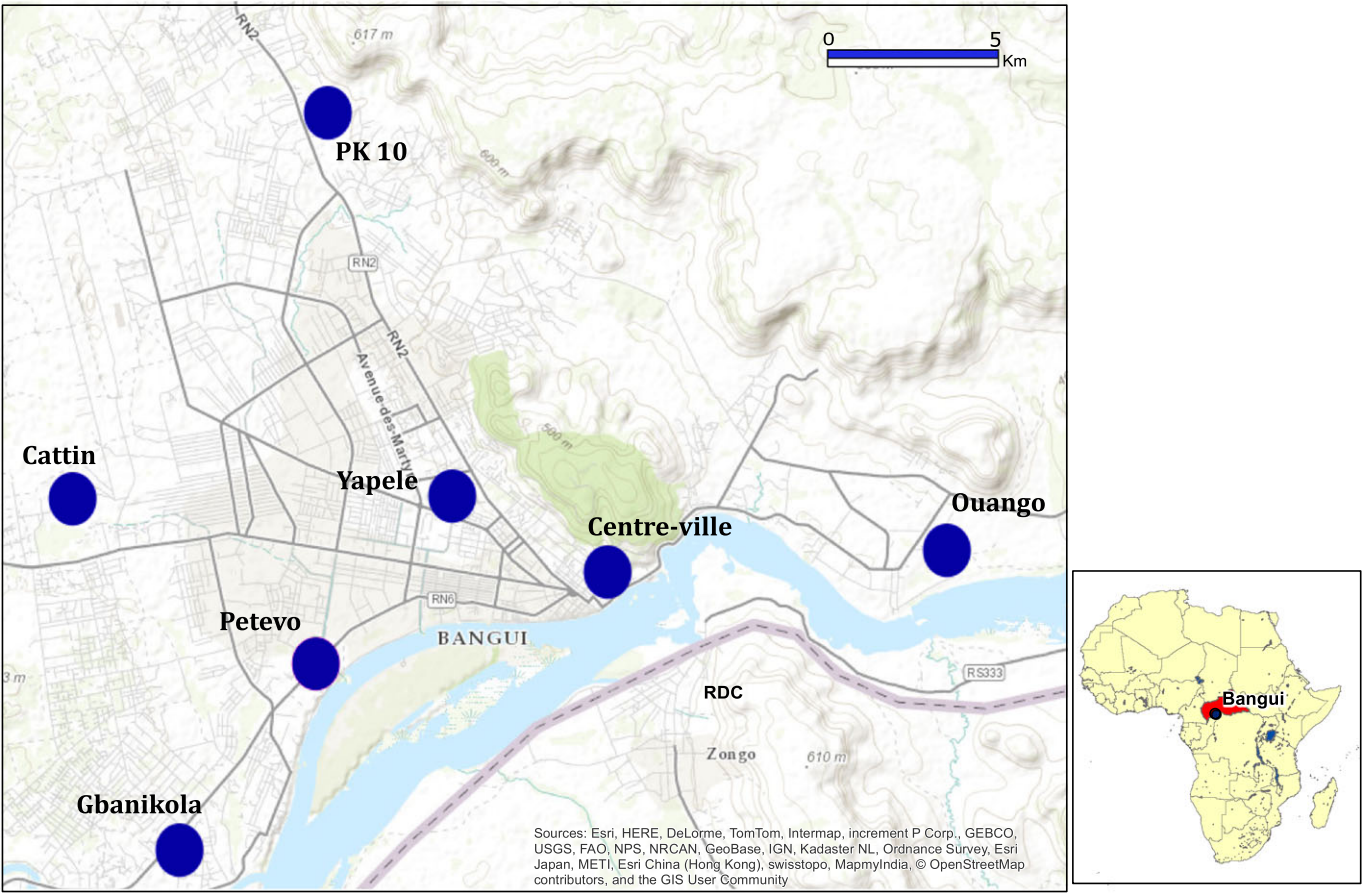
Map of Bangui (Central African Republic) area showing the seven districts where anopheline mosquitoes were collected

The study was carried out in 7 districts of Bangui (4°21′41″N, 18°33′19″E), the capital city of CAR: Ouango, Gbanikola, Petevo, Centre-ville, Yapele, PK10 and Cattin (Fig. 1). Bangui is bounded to the south by the Ubangi River, which borders the Democratic Republic of Congo (DRC), to the west by the municipality of Bimbo, the north by the town of Begoua (PK 12) and the east by Landja. The population was estimated at 839,081 inhabitants in 2012 (https://www.populationdata.net/pays/republique-centrafricaine/). Malaria transmission is perennial; all regions of the country are exposed to endemic malaria, with a peak during the rainy season. The average annual temperature is around 26 °C, with an annual precipitation of 1,510 mm. The study sites have been described in detail elsewhere [2, 16].

### Mosquito collection

Immature stages of mosquitoes were collected from different sites in 7 districts in Bangui during the rainy season between September to November 2014. The larval sampling was carried out on 12 breeding sites per district (with the minimum number of larvae per breeding site being 10) to minimize the loss of genetic information related to the potential isolation of breeding sites. Larvae from each district were pooled, fed Tetramin® baby fish food and kept under ambient conditions. Pupae were collected and placed in a mosquito cage covered with mosquito gauze and provided with a cotton sleeve for easy access to 10% sugar on filter paper. Adult mosquitoes were identified using the morphological identification keys of Gillies & De Meillon [18]. *Anopheles gambiae* were used for susceptibility tests, and a subset was further identified and genotyped to assess the mechanisms of resistance.

### Susceptibility assays

Non blood-fed, 2–3-day-old female *A. gambiae* mosquitoes grown from collected larvae were used for insecticide susceptibility tests. Bioassays were carried out using WHO test kits for adults mosquitoes [19], to assess the level of sensitivity (or resistance) of mosquitoes to insecticides. Six insecticides of technical grade quality were used: 2 pyrethroids (lambda-cyhalothrin 0.05%, deltamethrin 0.05%), 1 organochlorine (dichlorodiphenyltrichloroethane) (DDT 4%), 2 organophosphate (malathion 5%, fenitrothion 1%) and 1 carbamate (bendiocarb 0.1%). Impregnated papers were obtained from WHO reference center (Vector Control Research Unit, University Sains Malaysia, Penang, Malaysia). By district, 4 batches of 25 females were exposed to the diagnostic doses of insecticide treated papers for 60 min at 27 ± 1 °C and 80% relative humidity. The number of knockdown (KD) mosquitoes was recorded at 10, 15, 20, 30, 40, 50 and 60 min. After exposure, mosquitoes were kept in observation tubes and supplied with a 10% sugar/water solution. Expression of final mortality was measured 24 h after exposure. The *A. gambiae* Kisumu susceptible strain was used as a positive control. Mosquitoes exposed to untreated papers were used as the negative control. After the bioassays were completed, the mosquito specimens were individually stored in microcentrifuge tubes containing silica gel and stored at -20 °C for further molecular analysis.

### Biochemical analyses

A subset of *A. gambiae* not exposed to insecticides, and stored to -80 °C, was used for biochemical enzyme assays. Biochemical tests were carried out in all districts except Cattin for safety reasons. Activity levels of cytochrome P450, non-specific esterases (NSE) and glutathione S- transferases (GST) were calculated according the protocol described by Brogdon et al. [14], and modified by Fonseka-Gonzalez et al. [20]. Briefly, detoxifying enzyme activities were measured on single mosquitoes (*n* = 25) from each locality and stored at -80 °C within 24 h from emergence. Each mosquito was ground on ice in 200 μl of distilled water and the homogenate was centrifuged at 14,000× rpm for 2 min. Two 10 ml replicates of supernatant were transferred into 2 adjacent wells of a microtiter plate for NSE and GST analysis. Cytochrome P450 assays were performed with 2 × 20 ml replicates of supernatant.

### Cytochrome P450

Cytochrome P450 activity was determined using the heme-peroxidase assay to detect the elevation in the amount of heme, which is then converted into equivalent units of cytochrome P450. Eighty ml of 0.625 M potassium phosphate buffer, pH = 7.2 (Sigma P-5379) were added to 20 ml of mosquito homogenate together with 200 ml of tetramethyl benzidine solution (0.011 g 3,3’,5,5’tetramethyl Benzidine (Sigma T-8768) in 5 ml of 70% methanol (Sigma 32213) and 15 ml of 0.25 M sodium acetate buffer, pH = 5.0 (Sigma S-7899); 25 ml of 3% hydrogen peroxide (Sigma H-1009) was then added and the mixture incubated for 30 min at room temperature. Absorbance was read at 630 nm after 5 min incubation and values calculated from a standard curve of cytochrome C (Sigma C-7752).

### Non-specific esterases

Non-specific esterases activity was measured using α-Naphtol acetate (αNa) (Sigma N-8505) and β-Naphtol acetate (βNa) (Sigma N-6875). In each replicate well, 90 ml of phosphate buffer (PBS, pH = 6.5) and 100 ml of 0.6 M αNa (or βNa) were added to 10 ml of centrifuged mosquito homogenate. After 30 min incubation, 100 ml of Fast Garnett BC solution (8 g Fast Garnett Salt (Sigma F-8761) and 10 ml distilled water) was added to stop the reaction. The concentration of the final product was determined at 550 nm as an endpoint calculated from standard curves of α- (Sigma N-1000) and β-Naphtol (Sigma N-1250), respectively.

### Glutathione-S-transferases

To measure GST activity in mosquitoes, 200 ml of GSH/CDNB (Sigma G-6529) working solution (100 ml of an extemporaneous solution of 0.6% weight/volume reduced glutathione in 0.1 M sodium phosphate buffer pH = 6.5 and 0.013 g of 1-chloro-2,4 dinitrobenzene (Sigma C-6396) diluted in 1 ml of 70% methanol) were added to each replicate of mosquito homogenate. The reaction was read at 340 nm immediately as a kinetic assay for 5 min. An extinction coefficient of 5.76 mM^-1^ (corrected for a path length of 0.6 cm) was used to convert absorbance values to moles of product. Glutathione-S-transferases specific activity was reported as the rate of formation of GSH produced in mmol.min^-1^.mg^-1^ protein.

Absorbance was measured using a spectrophotometer type “Multiskan FC and Skanit Software” (www.thermo.com/readingroom) and the adjusted enzymatic mean activity of field *Anopheles* mosquitoes was compared to the Kisumu susceptible strain (originated from Kenya).

### DNA extraction and PCR amplification

DNA was extracted from individual mosquitoes using DNAzol essentially according to the manufacturer’s recommendations (Invitrogen, CA, USA). The total genomic DNA from each mosquito was re-suspended in 100 μl H_2_O and stored at -20 °C until use. For each site, a sample of 30 specimens was randomly selected, including the same number of dead and surviving specimens (when available) and used for molecular tests. Live and dead specimens of *A. gambiae* from the bioassay tests were subjected to the *A. gambiae* species specific PCR-RFLP assays for species identification according to the protocol of Fanello et al. [21]. The detection of knockdown resistance mutation L1014F (*kdr*-*w*) and L1014S (*kdr-e*) was used according to the protocols described by Martinez-Torres et al. [22] and Ranson et al. [23], respectively. A PCR-RFLP diagnostic test was used to detect the presence of insensitive acetylcholinesterase G119S mutation (Ace.1^*R*^ gene) according to the protocol of Weill et al. [24].

### Data analysis

WHO (2013) criteria were used to evaluate the resistance/susceptibility status of the tested mosquito populations (98–100% = susceptible and < 98% = resistance) [19]. The knockdown effect (KD) on tested mosquitoes was compared using the Kruskal-Wallis and Mann-Whitney tests. Mortality rates (expressed as a percentage of the number of dead mosquitoes by the total number of exposed mosquitoes) were compared using Fisher’s exact test. The allelic frequencies of L1014F, L1014S and G119S mutations were analysed to assess the variability in the frequency of mutations across populations. Biochemical assay data activities (enzymatic activity per mg of protein) of *A. gambiae* populations were compared to the Kisumu reference strain by Kruskal-Wallis and Mann-Whitney tests. Statistical analyses were performed using GraphPad Prism software v5.0 (www.graphpad.com). A *P*-value of 0.05 or less was considered as significant.

## Results

Molecular identification of a randomized sample (*n* = 210) indicated all specimens to be *A. gambiae* (Table 1). The S-form and M-form mosquitoes have been recently renamed as *A. gambiae* and *A. coluzzii*, respectively.

**Table 1:** Frequency of L1014 F (*kdr*-*w*) and L1014S (*kdr*-*e*) mutations

Districts	Total no. of samples tested	Species		Allelic profiles					Kdr frequency
		*A. coluzzii*	*A. gambiae*	*RwRw*	*RwS*	*ReRe*	*ReS*	*SS*	F_*kdr-w*_ (%)
Gbanikola	30	0	30	30	0	0	0	0	100
Pk 10	30	0	30	28	2	0	0	0	96.6
Ouango	30	0	30	29	1	0	0	0	98.3
Cattin	30	0	30	30	0	0	0	0	100
Centre-ville	30	0	30	26	4	0	0	0	93.3
Yapele	30	0	30	30	0	0	0	0	100
Petevo	30	0	30	30	0	0	0	0	100
Total	210	0	210	203	7	0	0	0	98.3

*Abbreviations*: *RwRw* homozygote resistant alleles west; *RwS* heterozygote resistant alleles west and susceptible; *ReRe* homozygote resistant alleles east; *ReS* heterozygote resistant alleles east and susceptible; *SS* homozygote susceptible alleles

A total of 224 tests were carried out during this study with 6 insecticides at the diagnostic doses according to the standard WHO protocol [19] (Additional file 1: Table S1). The mortality rate of the *A. gambiae* Kisumu susceptible strain, used as a positive control, was 100% for all tested insecticides. In the negative control, mortality rates were below 5%. Wild populations of *A. gambiae* from the seven sites surveyed in Bangui showed high resistance to pyrethroids and DDT.

### Susceptibility to pyrethroids

**Fig. 2:**
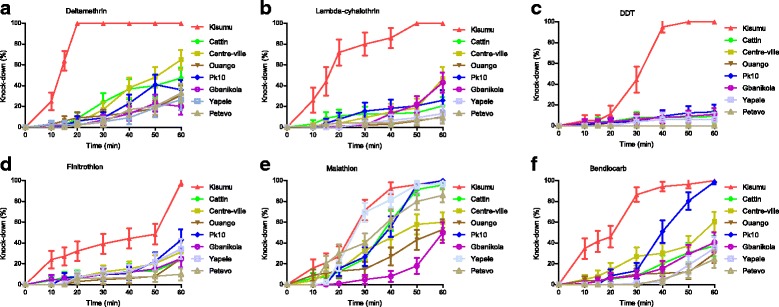
Effect of knockdown (KD) in *Anopheles gambiae* populations collected from seven districts in Bangui (Central African Republic) to 60 min exposure to **a** deltamethrin (0.05%), **b** lambda-cyhalothrin (0.05%), **c** DDT (4%), **d** fenitrothion (1%), **e** malathion (5%) and **f** bendiocarb (0.1%). The data represent medians with 95% confidence intervals

The KD of pyrethroids on tested mosquitoes was low in all surveyed sites. After 15 min exposure to deltamethrin, the number of KD mosquitoes varied from 0 to 6% (*χ* ^2^ = 7.55, *df* = 7, *P* = 0.37) against 64% for the Kisumu strain (Fig. 2a). At the same time, the proportion of the number of KD mosquitoes ranged from 0 to 8% (*χ* ^2^ = 8.24, *df* = 7, *P* = 0.31) for lambda-cyhalothrin against 44% for the Kisumu strain (Fig. 2b). After 30 min exposure, the proportion of KD mosquitoes for deltamethrin varied between 5 to 23% (*χ* ^2^ = 9.10, *df* = 7, *P* = 0.24) against 100% for the Kisumu strain. Meanwhile for lambda-cyhalothrin, this number varied from 2 to 15% according districts (*χ* ^2^ = 14.10, *df* = 7, *P* = 0.049). At the same time the number of KD mosquitoes in the Kisumu strain, was 100 and 80% for deltamethrin and lambda-cyhalothrin respectively (Fig. 2a, b). After 1 h exposure, the number of KD mosquitoes ranged from 23 to 65% for deltamethrin and from 9 to 46% for lambda-cyhalothrin. No significant difference was observed between districts with deltamethrin (*χ* ^2^ = 12.65, *df* = 6, *P* = 0.081) unlike lambda-cyhalothrin where a significant difference was observed (*χ* ^2^ = 18.44, *df* = 6, *P* = 0.010). Gbanikola and Yapele showed a low KD effect after 1 h exposure with a no significant differencees for deltamethrin (Mann-Whitney *U* = 28.5, *Z* = -0.36, *P* = 0.75) and for lambda-cyhalothrin (Mann-Whitney *U* = 28.5, *Z* = -0.52, *P* = 0.75) (Fig. 2a, b).

**Fig. 3:**
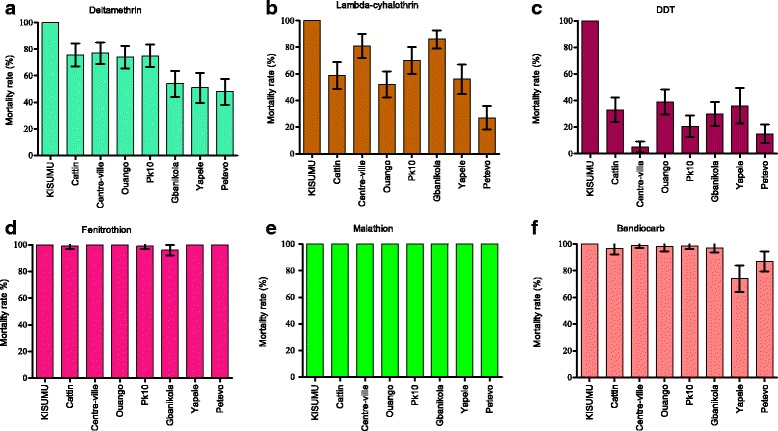
Mortality rates in *Anopheles gambiae* populations collected from seven districts in Bangui (Central African Republic) 24-h post-exposure to **a** deltamethrin (0.05%), **b** lambda-cyhalothrin (0.05%), **c** DDT (4%), **d** fenitrothion (1%), **e** malathion (5%) and **f** bendiocarb (0.1%). The data represent medians with 95% confidence intervals

The mortality rate (MR) of *A. gambiae* Kisumu susceptible strain, used as a control was 100% for all tested insecticides confirming the quality of the impregnated papers (Additional file 1: Table S1). The mean MR induced by deltamethrin in the wild *A. gambiae* populations from all districts was 71.4% (CI: 68.1–74.6). A significant difference of MR was observed according districts (*χ* ^2^ = 45.72, *df* = 6, *P* < 0.0001). The maximum was observed in Centre-ville with a MR of 77% (CI: 67.5–84.8) and the minimum in Petevo with a MR of 48% (CI: 37.9–58.2) (Fig. 3a).

Regarding lambda-cyhalothrin, the same trend was observed. The average MR in all districts was 63.4% (59.6–67.1). It was low in Petevo 27% (CI: 19–37.5) and the maximum was observed in Gbanikola 86% (CI: 77.6–92.1) (Fig. 3b). A significant difference of MR was observed between Petevo and Mbanikola (*χ* ^2^ = 69.02, *df* = 1, *P* < 0.0001).

### Susceptibility to DDT

The KD proportion to DDT was very low in comparison to the other tested insecticides. Fifteen minutes after exposure, the number of KD mosquitoes ranged from 0 to 4% in the wild *A. gambiae* populations and 6% in the Kisumu strain (Fig. 2c). No significant difference of KD mosquitoes was observed according districts (*χ* ^2^ = 10.3, *df* = 6, *P* = 0.172) whereas a significant difference of KD was observed after 30 min of exposure (*χ* ^2^ = 18.2, *df* = 6, *P* = 0.011). The same observation was made after 60 min of exposure where the number of KD mosquitoes ranged from 0 to 13.4% *versus* 100% in the Kisumu strain (*χ* ^2^ = 30.42, *df* = 6, *P* < 0.0001).

The average MR after 24 h in all districts was 25.4% (CI: 22.2–28.8) and varied according districts. A significant difference of MR was observed according districts (*χ* ^2^ = 48.52, *df* = 6, *P* < 0.0001). The low MR was observed in Centre-ville (5%, CI: 1.6–11.3) and the highest in Ouango (39%, CI: 29.4–49.3) (Fig. 3c).

### Susceptibility to organophosphates (OP)

In all districts, the number of KD mosquitoes after 1 h exposure was very low, ranging from 10% in Petevo to 43% in PK10 (Fig. 2d). At the same time 97% of KD mosquitoes was observed in the Kisumu strain. However, no significant difference of KD mosquitoes was observed according districts (*χ* ^2^ = 5.4, *df* = 6, *P* = 0.48). By contrast, a full susceptibility to fenitrothion was observed in all districts except Gbanikola where a MR of 96% was observed (Fig. 3d).

After 60 min exposure to malathion, the number of KD mosquitoes ranged from 50% in Gbanikola to 100% in PK10. At the same time 100% of KD mosquitoes was observed in the Kisumu strain. No significant difference of KD mosquitoes was observed according districts (*χ* ^2^ = 7.81, *df* = 6, *P* = 0.34) (Fig. 2e). Meanwhile, 100% of MR was observed in all districts after 24 h (Fig. 3e).

### Susceptibility to bendiocarb

After 15 min of exposure, the KD effect induced by bendiocarb on the *A. gambiae* populations from the 7 Bangui districts ranged from 0 to 8% compared to 41% in the Kisumu strain. No significant difference of KD mosquitoes was observed across the study sites (*χ* ^2^ = 10.26, *df* = 6, *P* = 0.17). The proportion of KD mosquitoes after 60 min exposure ranged from 24% in Petevo to 98% in PK10 with a no significant difference according districts (*χ* ^2^ = 8.29, *df* = 6, *P* = 0.21). In addition, no difference of KD mosquitoes was observed between Petevo and PK10 (Mann-Whitney *U* = 19, *Z* = -1.36, *P* = 0.18). At the same time 100% of KD mosquitoes was observed in the Kisumu strain (Fig. 2f).

The mortality induced by bendiocarb was very heterogeneous. After 24 h, full susceptibility was observed in Centre-ville, PK10 and in Ouango with a MR ranging from 98 to 99%. A suspicion resistance was observed in Cattin and Gbanikola districts with a MR of 94.7% (CI: 89.5–99.5) and 97% (CI: 94.2–100), respectively. While a confirmed resistance was observed in Yapele and Petevo districts with a MR of 75% (CI: 62.8–82.8) and 87% (CI: 72.3–90.3), respectively (Fig. 3f).

### Molecular form identification and genotyping of *kdr* and *Ace-1* mutations

The detection of the *kdr* insecticide resistance allele L1014F frequency (*kdr-w* type) in a subsample of 210 *A. gambiae* revealed a high prevalence of this mutation, conferring that the variant is present in the voltage-gated sodium channel encoding *para* gene. Thus, more than 96.6% of the *A. gambiae* populations presented a homozygous resistance profile of the type RR (*n* = 203) and 3.3% a heterozygous profile RS (*n* = 7) (Table 1). None of the tested *A. gambiae* mosquitoes was found to be homozygous for the insecticide sensitive wild type *kdr* allele 1014 L. The allele *kdr-w* frequencies ranged from 93.3 to 100% depending on the district. No significant difference was observed between sites (*P* = 0.11, OR = 10.36, CI: 0.53–201.6). However, no L1014S (*kdr-e*) and *Ace-1* ^*R*^ mutation was observed among the tested samples.

### Biochemical assays

**Fig. 4:**
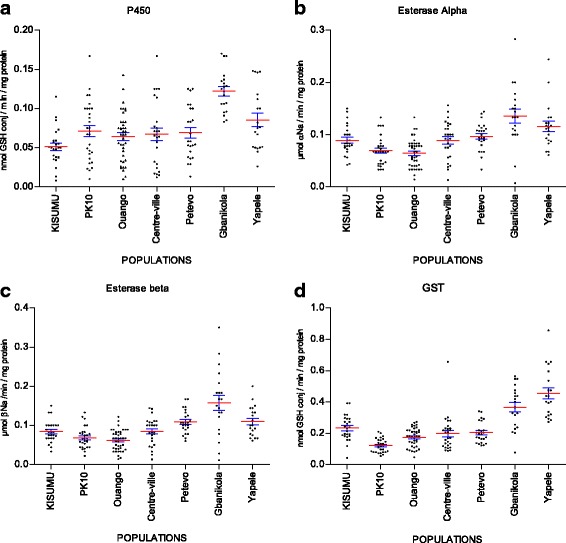
Detoxifying enzyme activities in *Anopheles gambiae* populations collected form seven districts in Bangui (Central African Republic) in comparison with Kisumu susceptible strain. **a** Cytochrome P450 activities (MFO). **b** Alpha esterase activities. **c** Beta esterase activities. **d** Glutathione S-transferases activities (GST). *Red* lines represent means with 95% confidence intervals (*blue lines*)

The average levels of enzymatic activities of cytochrome P450, esterases and GST compared to the Kisumu susceptible strain are shown in Fig. 4. Biochemical assays showed high level enzymatic activities of the cytochrome P450, NSE and GST by district.

The average level of P450 activity (MFO) in *A. gambiae* tested in the different sites is shown in Fig. 4a. The activity of cytochrome P450 was significantly higher in Gbanikola and Yapele compared to the Kisumu strain (*χ* ^2^ = 31.85, *df* = 2, *P* < 0.0001). By contrast, no significant difference of this activity of cytochrome P450 was observed between Petevo, PK 10, Ouango and Centre-ville compared to the Kisumu strain (*χ* ^2^ = 5.08, *df* = 4, *P* = 0.27).

Overexpression of GST activity was observed in the populations of *A. gambiae* from Ouango, Centre-ville, Gbanikola and Yapele compared to the Kisumu strain (*χ* ^2^ = 63.16, *df* = 5, *P* < 0.0001). Furthermore, the level of GST activity did not reveal any significant differences between Kisumu and Petevo (Mann-Whitney *U* = 231, *Z* = -1.38, *P* = 0.17). By contrast, in Pk10, the GST activity was significantly lower compared to the Kisumu strain (Mann-Whitney *U* = 73.5, *Z* = -5.01, *P* < 0.0001) (Fig. 4d).

A significantly increased esterase activity (using α-naphthyl acetate) was observed in the *A. gambiae* populations from Gbanikola and Yapele compared to the Kisumu susceptible strain (*χ* ^2^ = 13.95, *df* = 2, *P* = 0.0009). By contrast, α-esterase activity was significantly low in *Anopheles* populations from PK10 and Ouango compared to Kisumu (*χ* ^2^ = 11.50, *df* = 2, *P* = 0.003). However, no significant difference was observed in the *A. gambiae* populations from Centre-ville and Petevo compared to Kisumu (*χ* ^2^ = 1.32, *df* = 2, *P* = 0.51) (Fig. 4b).

The activity of NSE (using β naphtyl acetate as a substrate) was higher in the *A. gambiae* populations from Gbanikola, Petevo and Yapele compared to the Kisumu strain (*χ* ^2^ = 17.34, *df* = 3, *P* = 0.0006). In contrast, the β-esterase activity was significantly low in mosquitoes from PK10 and Ouango in comparison to the Kisumu strain (*χ* ^2^ = 8.81, *df* = 2, *P* = 0.012). No significant difference was observed in mosquitoes from Centre-ville compared to Kisumu (Mann-Whitney *U* = 307.5, *Z* = −0.54, *P* = 0.58) (Fig. 4c).

## Discussion

This study, conducted in seven districts of Bangui allows us to report, for the first time in CAR, the level of susceptibility of the main malaria vector, *A. gambiae,* to the different families of insecticides conventionally used in vector control. Our study revealed that *A. gambiae* population from Bangui are resistant to DDT and pyrethroids with a high prevalence of the *kdr-w* mutation; on the other hand, the *kdr-e* mutation was not identified in any tested mosquitoes. The presence of *kdr* mutations have been studied all around Africa [25]. Previously, kdr-w mutation was observed only in West Africa, whereas it now appears to be invading East and Central Africa, with the direct consequence of barrier disappearance between kdr-e and kdr-w, allowing significant gene flow among different anopheles populations [26, 27]. Also, a moderate resistance to bendiocarb and a full susceptibility to organophosphates have been observed.

Despite the absence of the *Ace-1* ^*R*^ mutation, this study showed the emerging resistance in the *A. gambiae* population*s* from Yapele to bendiocarb, a first in CAR. This decreased susceptibility of *A. gambiae* populations to bendiocarb can be explained by the use of carbamate-based insecticide sprays inside houses and carbamate-based pesticides for agricultural purposes more so as this area is known to be highly agricultural. However, the emerging resistance of *A. gambiae* populations to bendiocarb had already been reported in many African countries such as Nigeria [28], Benin [29, 30], Guinea Conakry [31] and Congo [32], and therefore is not a new phenomenon.

Surprisingly no *Ace-1* ^*R*^ mutation has been found in *A. gambiae* populations from Bangui, despite the emerging bendiocarb resistance suggesting the involvement of metabolic resistance. Biochemical analysis measuring the enzymatic activity of cytochrome P450, esterases and GST of the Kisumu strain and *A. gambiae* populations showed that cytochrome P450 activity was significantly higher in *A. gambiae* than the Kisumu strain in all seven districts. Our data clearly indicates that the pyrethroid resistance in Bangui is driven by the co-existence of cytochrome P450 and *kdr*. Furthermore, a significant overexpression activity of GST and NSE (alpha and beta) in comparison to the Kisumu susceptible strain was observed in some districts, particularly in Gbanikola and Yapele, suggesting a multiple resistance including *kdr* and GST in the resistance of DDT. Accordingly, these data can justify the slow effect of fenitrothion on mosquito populations in the absence of the *Ace-1* ^*R*^ mutation. The same results have been observed in Cameroon by Nwane et al. [15] and in Benin by Assogba et al. [13].

The evidence of resistance of *A. gambiae* to the two major classes of insecticides (pyrethroids and DDT) in Bangui is alarming and constitutes a potential threat to the success of malaria vector control programmes. In our study, a significance difference of insecticide resistance was observed between districts. This indicates that there are potentially different levels of selection across the city, possibly due to differences in bednet usage, IRS rates and the use of pyrethroids as pesticides, which have all been recognized as factors responsible for the selection of resistant mosquitoes in sub-Saharan Africa [6, 33].

The presence of *A. gambiae* in all specimens tested is a clear indication of the dominance of this species in this region and is in agreement with the rest of CAR [16]. The same results have been observed in Equatorial Guinea [34], Gabon [35], Chad [36] and in the Democratic Republic of Congo [37]. However in Cameroon, a predominance of *A. coluzzii* was observed [34].

Strong resistance to insecticides is a constraint for many reasons. Recent studies have shown that *kdr*-type resistance could seriously compromise the effectiveness of insecticide-treated nets [6, 8], and the presence of *kdr* mutations in *Anopheles* may significantly increase their susceptibility to *Plasmodium* infection [38]. The delicacy of this situation in the capital Bangui should lead to further investigation in other parts of the country through both the exploration of malaria vectors’ resistance profile in sentinel sites and the mapping of resistance in CAR. This will provide information for the design of adequate measures to anticipate and manage the resistance phenomenon. For that purpose, a national survey on pesticides commonly used in CAR has been recommended to develop knowledge of the local factors that drive resistance selection.

Finally, this study shows the presence of multiple resistance mechanisms in *A. gambiae* populations, which is not without consequence in the future development of vector control strategies especially for targeted malaria control [17]. Hence the choice of future insecticide in vector control by CAR national malaria control programme must be reexamined in view of these results.

## Conclusion

This study showed, for the first time in CAR, molecular and biological evidence of resistance to pyrethroids and DDT in *A. gambiae* populations from Bangui including target-site mutation and metabolic mechanism sustained by the early development of resistance to carbamates. The co-existence of these resistance mechanisms in *A. gambiae* constitute serious obstacle for the future success of malaria control programmes based on ITNs and IRS.

## Additional file

Additional file 1: Table S1. Number of *Anopheles* (%) tested by bioassays in seven sites of Bangui, Central African Republic by using WHO test kits for adult mosquitoes. Six insecticides of technical grade were used, including two pyrethroids (deltamethrin and lambda-cyhalothrin), one carbamate (bendiocarb), two organophosphates (fenitrothion and malathion) and one organochlorine (DDT). (XLSX 15 kb)

## Abbreviations

ACT: Artemisinin based combination therapy
CAR: Central African Republic
DDT: Dichlorodiphenyltrichloroéthane
GST: Glutathione S- transferases
IRS: Indoor residual spraying
ITN: Insecticide-treated nets
KD: Knockdown
MR: Mortality rate
NSE: Non-specific esterases (NSE)
RDTs: Rapid diagnostic tests
WHO: World Health Organization

## References

[1] MSF. Rapport Médecins Sans Frontières 2011. Une Crise silencieuse République Centrafricaine; http://www.msf.fr/actualite/publications/rapport-republique-centrafricaine-crise-silencieuse. Accessed 13 Dec 2011.

[2] Sangba ML, Deketramete T, Wango SP, Kazanji M, Akogbeto M, Ndiath MO (2016). Insecticide resistance status of the *Anopheles funestus* population in Central African Republic: a challenge in the war. Parasit Vectors.

[3] Rapport ministère de la santé de la République Centrafricaine 2015. Plan de transition du secteur santé 2015–2016; http://www.nationalplanningcycles.org/sites/default/files/planning_cycle_repository/central_african_republic/rca_-ptss_v_definitive_1.pdf. Accessed 14 Feb 2015.

[4] MSF. Rapport Médecins Sans Frontières 2015. Crise en République Centrafricaine et réfugiés dans les pays frontaliers; http://www.msf.fr/actualite/dossiers/republique-centrafricaine-crise-silencieuse. Accessed 16 June 2016.

[5] Padonou GG, Sezonlin M, Razaki O, Aizoun N, Oké-Agbo F, Ousou O (2012). Impact of three years of large scale Indoor Residual Spraying (IRS) and Insecticide Treated Nets (ITNs) interventions on insecticide resistance in *Anopheles gambiae* s.l. in Benin. Parasit Vectors.

[6] Trape JF, Tall A, Diagne N, Ndiath O, Ly AB, Faye J (2011). Malaria morbidity and pyrethroid resistance after the introduction of insecticide-treated bednets and artemisinin-based combination therapies: a longitudinal study. Lancet Infect Dis.

[7] Enayati A, Hemingway J (2010). Malaria management: past, present, and future. Annu Rev Entomol.

[8] Corbel V, Akogbeto M, Damien GB, Djenontin A, Chandre F, Rogier C (2012). Combination of malaria vector control interventions in pyrethroid resistance area in Benin: a cluster randomised controlled trial. Lancet Infect Dis.

[9] Nkya TE, Poupardin R, Laporte F, Akhouayri I, Mosha F, Magesa S (2014). Impact of agriculture on the selection of insecticide resistance in the malaria vector *Anopheles gambiae*: a multigenerational study in controlled conditions. Parasit Vectors.

[10] Ranson H, Lissenden N (2016). Insecticide resistance in African *Anopheles* mosquitoes. A worsening situation that needs urgent action to maintain malaria control. Trends Parasitol.

[11] Knox TB, Juma EO, Ochomo EO, Jamet HP, Ndungo L, Chege P (2014). An online tool for mapping insecticide resistance in major *Anopheles* vectors of human malaria parasites and review of resistance status for the Afrotropical region. Parasit Vectors.

[12] Nwane P, Etang J, Chouaibou M, Toto JC, Koffi A, Mimpfoundi R (2013). Multiple insecticide resistance mechanisms in *Anopheles gambiae* s.l. populations from Cameroon, Central Africa. Parasit Vectors.

[13] Assogba BS, Djogbénou LS, Saizonou J, Milesi P, Djossou L, Djegbe I (2014). Phenotypic effects of concomitant insensitive acetylcholinesterase (ace-1(R)) and knockdown resistance (kdr (R)) in *Anopheles gambiae*: a hindrance for insecticide resistance management for malaria vector control. Parasit Vectors.

[14] Brogdon WG, McAllister JC, Vulule J (1997). Heme peroxidase activity measured in single mosquitoes identifies individuals expressing an elevated oxidase for insecticide resistance. J Am Mosq Control Assoc.

[15] Nwane P, Etang J, Chouasmallyi UM, Toto JC, Koffi A, Mimpfoundi R, Simard F (2013). Multiple insecticide resistance mechanisms in *Anopheles gambiae* s.l. populations from Cameroon, Central Africa. Parasit Vectors.

[16] Ndiath MO, Eiglmeier K, Ole-Sangba ML, Holm I, Kazanji M, Vernick KD (2016). Composition and genetics of malaria vector populations in the Central African Republic. Malar J.

[17] Chihanga S, Haque U, Chanda E, Mosweunyane T, Moakofhi K, Jibril HB (2016). Malaria elimination in Botswana, 2012–2014: achievements and challenges. Parasit Vectors.

[18] Gillies MT, De Meillon D (1968). The Anophelinae of Africa South of the Sahara. Publ South Afri Inst Med Res.

[19] WHO. World Health Organization (2013). Test procedures for insecticide resistance monitoring in malaria vector mosquitoes.

[20] Fonseca-Gonzalez I, Quinones ML, McAllister J, Brogdon WG (2009). Mixed-function oxidases and esterases associated with cross-resistance between DDT and lambda-cyhalothrin in *Anopheles darlingi* Root 1926 populations from Colombia. Mem Inst Oswaldo Cruz.

[21] Fanello C, Santolamazza F, della Torre A (2002). Simultaneous identification of species and molecular forms of the *Anopheles gambiae* complex by PCR-RFLP. Med Vet Entomol.

[22] Martinez-Torres D, Foster SP, Field LM, Devonshire AL, Williamson MS (1999). A sodium channel point mutation is associated with resistance to DDT and pyrethroid insecticides in the peach-potato aphid, *Myzus persicae* (Sulzer) (Hemiptera: Aphididae). Insect Mol Biol.

[23] Ranson H, Jensen B, Vulule JM, Wang X, Hemingway J, Collins FH (2000). Identification of a point mutation in the voltage-gated sodium channel gene of Kenyan *Anopheles gambiae* associated with resistance to DDT and pyrethroids. Insect Mol Biol.

[24] Weill M, Malcolm C, Chandre F, Mogensen K, Berthomieu A, Marquine M (2004). The unique mutation in ace-1 giving high insecticide resistance is easily detectable in mosquito vectors. Insect Mol Biol.

[25] Ranson H, N’Guessan R, Lines J, Moiroux N, Nkuni Z, Corbel V (2011). Pyrethroid resistance in African anopheline mosquitoes: what are the implications for malaria control?. Trends Parasitol.

[26] Ndiath MO, Cailleau A, Orlandi-Pradines E, Bessell P, Pages F, Trape JF (2015). Emerging knock-down resistance in *Anopheles arabiensis* populations of Dakar, Senegal: first evidence of a high prevalence of kdr-e mutation in West African urban area. Malar J.

[27] Antonio-Nkondjio C, Tene Fossog B, Kopya E, Poumachu Y, Menze Djantio B, Ndo C (2015). Rapid evolution of pyrethroid resistance prevalence in *Anopheles gambiae* populations from the cities of Douala and Yaounde (Cameroon). Malar J.

[28] Oduola AO, Idowu ET, Oyebola MK, Adeogun AO, Olojede JB, Otubanjo OA (2012). Evidence of carbamate resistance in urban populations of *Anopheles gambiae* s.s. mosquitoes resistant to DDT and deltamethrin insecticides in Lagos, South-Western Nigeria. Parasit Vectors.

[29] Aizoun N, Aikpon R, Gnanguenon V, Oussou O, Agossa F, Padonou G (2013). Status of organophosphate and carbamate resistance in *Anopheles gambiae* sensu lato from the south and north Benin, West Africa. Parasit Vectors.

[30] Djogbenou L, Dabire R, Diabate A, Kengne P, Akogbeto M, Hougard JM (2008). Identification and geographic distribution of the ACE-1R mutation in the malaria vector *Anopheles gambiae* in south-western Burkina Faso, West Africa. Am J Trop Med Hyg.

[31] Vezenegho SB, Brooke BD, Hunt RH, Coetzee M, Koekemoer LL (2009). Malaria vector composition and insecticide susceptibility status in Guinea Conakry, West Africa. Med Vet Entomol.

[32] Koekemoer LL, Spillings BL, Christian RN, Lo TC, Kaiser ML, Norton RA (2011). Multiple insecticide resistance in *Anopheles gambiae* (Diptera: Culicidae) from Pointe Noire, Republic of the Congo. Vector Borne Zoonotic Dis.

[33] Diabate A, Baldet T, Chandre F, Akoobeto M, Guiguemde TR, Darriet F (2002). The role of agricultural use of insecticides in resistance to pyrethroids in *Anopheles gambiae* s.l. in Burkina Faso. Am J Trop Med Hyg.

[34] Tene Fossog B, Ayala D, Acevedo P, Kengne P, Ngomo Abeso Mebuy I, Makanga B (2015). Habitat segregation and ecological character displacement in cryptic African malaria mosquitoes. Evol Appl.

[35] Mourou JR, Coffinet T, Jarjaval F, Pradines B, Amalvict R, Rogier C (2010). Malaria transmission and insecticide resistance of *Anopheles gambiae* in Libreville and Port-Gentil, Gabon. Malar J.

[36] Kerah-Hinzoumbe C, Peka M, Antonio-Nkondjio C, Donan-Gouni I, Awono-Ambene P, Same-Ekobo A (2009). Malaria vectors and transmission dynamics in Goulmoun, a rural city in south-western Chad. BMC Infect Dis.

[37] Trape JF (1987). Malaria and urbanization in central Africa: the example of Brazzaville. Part IV. Parasitological and serological surveys in urban and surrounding rural areas. Trans R Soc Trop Med Hyg.

[38] Ndiath MO, Cailleau A, Diedhiou SM, Gaye A, Boudin C, Richard V, Trape JF (2014). Effects of the kdr resistance mutation on the susceptibility of wild Anopheles gambiae populations to Plasmodium falciparum: a hindrance for vector control. Malar J.

